# Aging is associated with sex-specific alteration in the expression of genes encoding for neuroestradiol synthesis and signaling proteins in the mouse trigeminal somatosensory input

**DOI:** 10.1007/s11357-024-01268-z

**Published:** 2024-07-02

**Authors:** Álvaro Bautista-Abad, Nuria García-Magro, Daniel Pinto-Benito, Julio Eduardo Cáceres-Pajuelo, Carlos Vicente Alises, Danny Ganchala, Natalia Lagunas, Pilar Negredo, Luis Miguel García-Segura, Maria-Angeles Arevalo, Daniela Grassi

**Affiliations:** 1 Department of Anatomy, Histology and Neuroscience, Autonoma University of Madrid, Calle Arzobispo Morcillo 4, Madrid, Spain; 2 Facultad de Ciencias de la Salud, Universidad Francisco de Vitoria, Ctra. Pozuelo-Majadahonda Km 1,800, Pozuelo de Alarcón, Madrid, Spain; 3 Neuroactive Steroids Lab, Cajal Institute, CSIC, Avenida Doctor Arce 37, Madrid, Spain; 4 Centro de Investigación Biomédica en Red de Fragilidad y Envejecimiento Saludable (CIBERFES), Instituto de Salud Carlos III, Madrid, Spain; 5 Department of Legal Medicine, Psychiatry and Pathology, School of Medicine, Complutense University of Madrid, Plaza Ramón y Cajal s/n, Madrid, Spain

**Keywords:** Aging, Sex differences, Aromatase, Estrogen receptors, Androgen receptor, Trigeminal ganglion

## Abstract

Pain perception is influenced by sex and aging, with previous studies indicating the involvement of aromatase, the estradiol synthase enzyme, in regulating pain perception. Previous research has established the presence of aromatase in dorsal root ganglia sensory neurons and its role in modulating pain perception. The present study aims to explore the implications of aging and sex on the expression of aromatase and estrogen receptors in the trigeminal ganglion. The study examined mRNA levels of aromatase, ERs, and the androgen receptor (AR) in the trigeminal ganglion of 3-month-old and 27-month-old male and female mice, as well as 3-month-old mice from the four-core genotype (FCG) transgenic model. The latter facilitates the assessment of gonadal hormone and sex chromosome implications for sex-specific traits. Aromatase localization in the ganglion was further assessed through immunohistochemistry. Aromatase immunoreactivity was observed for the first time in sensory neurons within the trigeminal ganglion. Trigeminal ganglion gene expressions were detected for aromatase, ERs, and AR in both sexes. Aromatase, ERβ, and GPER gene expressions were higher in young males versus young females. Analyses of the FCG model indicated that sex differences depended solely on gonadal sex. The aging process induced an enhancement in the expression of aromatase, ERs, and AR genes across both sexes, culminating in a reversal of the previously observed gender-based differences. the potential impact of estrogen synthesis and signaling in the trigeminal ganglion on age and sex differences warrants consideration, particularly in relation to trigeminal sensory functions and pain perception.

**Graphical abstract:**

Age and gonadal sex influence ERs, AR, and ARO levels in the trigeminal ganglion. Although somatosensory perception shows a decline in the elderly, the incidence of trigeminal neuralgia intensifies in aged adults and is predominantly prevalent in women relative to men. The increased expression of aromatase and estrogen receptors in aged female animals suggests that the modulatory influence that neuroestradiol exerts over the trigeminal somatosensory input, inclusive of pain, undergoes changes in elderly male and female individuals in a sex-specific manner.
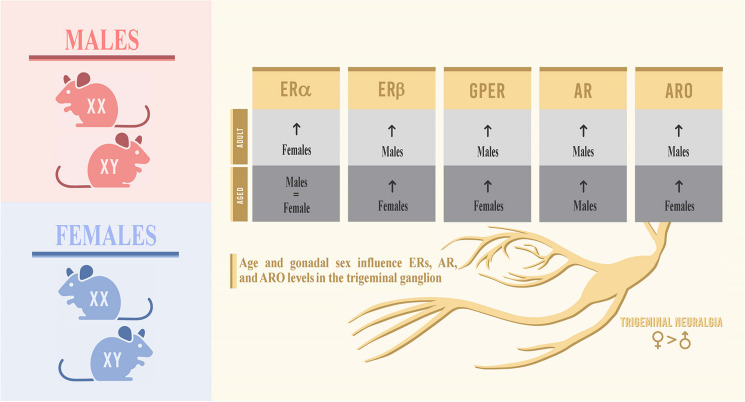

## Introduction

Although somatosensory perception shows a decline in older people [1], trigeminal neuralgia is more frequent in older adults [2, 3], in line with the higher prevalence of other chronic pain conditions in the elderly [4, 5].

In addition to cultural, cognitive, and psychosocial influences, biological sex is one of the factors that affect pain perception [5, 6]. Thus, chronic pain conditions [5], including trigeminal neuralgia [3, 7], are more common in women than men. However, the role of gonadal hormones in the generation of these sex differences is unclear [5].

Sex-related differences in pain perception have also been investigated in animal models, with studies generally reporting that female rodents exhibit increased sensitivity to noxious stimuli compared to males. Estradiol, depending on concentration and pain modality, may alleviate or potentiate hyperalgesia [8–10]. Estradiol actions on pain regulation are mediated through estrogen receptor (ER)α, ERβ and G protein-coupled ER (GPER, GPR30), which are expressed in nervous system structures involved in nociception [9, 11–14].

Estrogenic actions in the pain-processing neuronal circuits are in part driven by hormonal estradiol released by peripheral steroidogenic organs, such as the ovary. However, estradiol is also a neural-derived paracrine factor, or neuroestradiol, which is synthesized locally in the pain circuits in both sexes from circulating or local androgens by the enzyme aromatase or estrogen synthase [15–17].

Consistent with the pain symptoms often experienced by breast cancer patients treated with aromatase inhibitors [18], spinal or systemic administration of these molecules in rodents increases nociception and induces allodynia [19–21]. This evidence, combined with increased aromatase expression in the dorsal horn of the spinal cord under pain conditions [20] and the localization of aromatase and ERs in specific signaling domains involved in nociceptive regulatory signaling [22], suggests that neuroestradiol may activate antinociceptive mechanisms, possibly complementing nociceptive modulation exerted by systemic estradiol.

In this study, we first examined whether aromatase is expressed in the primary sensory neurons of the mouse trigeminal ganglion. Then, given the reported effects of aging and sex on pain perception in humans, we analyzed the expression of aromatase and ERs in the trigeminal ganglion of adult and older animals of both sexes. Furthermore, we used the Four Core Genotype (FCG) mouse model [23] to determine whether gonadal sex, sex chromosome complement, or a combination of both factors cause the observed sex differences.

## Methods

### Animals

Male and female CD1 wild-type and Four Core Genotype (FCG) mice from our in-house colony were maintained on a 12:12 hour light-dark cycle with ad libitum access to food and water. Mating involved placing a male in a cage with two females for one week. Pregnant females were individually housed in plastic maternity cages containing wood shavings as nesting material. The FCG transgenic model was generated by deleting the testis-determining gene Sry from the Y chromosome and inserting a Sry transgene into autosome 3 in male CD1 mice (FCG XYm mice). By mating FCG XYm mice with CD1 wild-type females, the resulting offspring included XX and XY mice with testes, as well as XX and XY mice with ovaries. This allowed for the comparison of XX and XY mice with the same type of gonad to assess the phenotypic effects of sex chromosome complement in cells and tissues. Genotyping of FCG mice was performed using RT-PCR to detect Sry and Ssty (located on the Y chromosome) gene transcripts. FCG model mice [24] were obtained from the Cajal Institute colony, which originated from animals donated by Dr. A.P. Arnold.

### Experimental procedure

Three-month-old CD1 wild-type male and female mice, along with FCG mice and 27-month-old male and female wild-type mice, were used in the study. Four animals per experimental group were decapitated, and their trigeminal ganglia were dissected under a magnifying glass. The samples were then rapidly frozen in dry ice and stored at -80°C until further processing. Three-month-old wild-type and FCG females were sacrificed during the diestrus 2 phase. The determination of the estrous cycle phase was performed by analyzing vaginal smears [25].

### Immunofluorescence

The detection of aromatase presence was executed through immunofluorescence performed on glass slide-mounted sections, adhering to the subsequent steps. Trigeminal ganglia, sliced at 15 μm, were directly placed on a glass slide in four adjacent series and preserved at room temperature until the immunofluorescence procedure was conducted. All steps were carried out at room temperature. Initially, sections underwent a 30-minute wash in PBS 0.1 M, pH 7.3–7.4, and nonspecific binding sites were obstructed by a 2-hour incubation in blocking solution (PBS 0.1 M, pH 7.3–7.4, 2% Triton X-100 (Merck, Darmstadt, Germany), and 10% normal donkey serum). Subsequently, sections were incubated overnight with the primary anti-aromatase antibody (diluted 1:1000 in blocking solution, in-house production, described and validated by [26]. The following day, sections were washed for 30 minutes in PBS 0.002 M, pH 7.3–7.4, and then incubated with the Alexa anti-rabbit 488 secondary antibody (Thermo Fisher Scientific, 1:200) for 2 hours. Lastly, sections were rinsed for 30 minutes in PBS 0.002 M, pH 7.3–7.4, and covered utilizing fluoromount DAPI. A Leica TCS SP5 Spectral confocal system (Leica Microsystems AG, Wetzlar, Germany) facilitated the observation of sections and the acquisition of microphotographs. Confocal images were collected using the z-stack function at a resolution of 1024 x 1024 pixels, employing 10X and 40X oil immersion objectives.

### Quantitative real-time polymerase chain reaction (PCR)

Total RNA was extracted from isolated tissues utilizing TRIzol reagent, adhering to the manufacturer's guidelines (Thermo Fisher Scientific) to quantitate *Cyp19a1*, which encodes the enzyme aromatase; *Esr1*, encoding for estrogen receptor (ER)α; *Esr2*, encoding for ERβ; *Gper1*, encoding for G-protein coupled ER (GPER); *Ar*, encoding for androgen receptor (AR); and *Rpl13a*, selected as the control housekeeping gene. The first cDNA strand was synthesized employing 1 μg of extracted RNA through reverse transcription within a final volume of 15 μL, using the RevertAid H Minus first-strand cDNA synthesis kit (M.B.I. Fermentas) following the manufacturer's guidelines. Upon reverse transcription, cDNA was diluted to 1:10 for *Cyp19a1*, *Esr1*, *Esr2,* and *Ar*, and 1:100 for housekeeping gene quantification. Subsequently, 5 μL per sample were amplified through real-time PCR in 20 μL. *Cyp19a1* amplification was executed with Taqman master mix and specific primers (Mm00484049_m1 Cyp19a1). The quantification of *Esr1*, *Esr2*, *Gper1*, *Ar,* and *Rpl13a* was undertaken using the SYBR Green master mix (Applied Biosystems), as previously delineated [27, 28] with the primers detailed in Table 1.

**Table 1:** Primers sequences. Sequences of the forward and revers primers used for *Esr1*, *Esr2*, *Gper1*, and *Ar* mRNA levels analysis by qPCR

Gene	Primer forward	Primer reverse
*Esr1*	5’-GATCCCACCATGCACAGTGA-3'	5’-GGAGCATCTACAGGAACACAGGTA-3'
*Esr2*	5’-CCTGGTCTGGGTGATTTCGA-3'	5’-ACTGATGTGCCTGACATGAGAAAG-3'
*Gper1*	5’- TGCTGCCATCCAGATTCAAG -3'	5’- GGGAACGTAGGCTATGGAAAGAA-3'
*Ar*	5’-TCTACTTTGCACCTGACTTGGTTT-3'	5’-ACTCTTGAGACAGGTGCCTCATC-3'
*Rpl13a*	5’-TACCAGAAAGTTTGCTTACCTGGG-3'	5’-TGCCTGTTTCCGTAACCTCAAG-3'

All samples were processed in duplicate utilizing an ABI Prism 7500 sequence detector (Applied Biosystems), adhering to conventional Applied Biosystems cycling parameters (40 cycles of 95 °C for 15 s, and 60 °C for 1 min). Following each PCR reaction, dissociation curve analysis was executed to ascertain the presence of a singular product and the absence of primer dimers. The Ct was ascertained and normalized to the housekeeping gene Rpl13a, employing the ΔΔCT method to determine relative expression levels. Statistical analyses were conducted using ΔΔCT threshold cycle values.

### Statistical Analysis

Statistical significance was evaluated using various methods: unpaired two-tailed t-test was used for examining differences between young adult and aged groups and two-way analysis of variance (ANOVA) for analyzing interactions between the independent variables "genotype" and "gonads" in FCG animals, as well as "sex" and "age" in young adult and aged female and male animals. Subsequently, for those variables/interactions for which ANOVA *p* values were statistically significant, the statistical significance of the effects of each independent variable and their interactions was tested by Fisher's post hoc comparisons if no interaction between independent variables was found or simple comparisons in the case of significant interaction. Simple linear regression analysis was employed to determine potential relationships between two distinct variables, with the calculation of the coefficient of determination (R2) and Spearman's correlation coefficient (r) values. Correlation analysis was employed to determine potential relationships between distinct variables, with the calculation of the coefficient of determination (R2), and data were plotted using the function ‘ggcorrplot’ Rstudio package, ggcorrplot (v0.1.4.1). Statistical analyses were executed utilizing R “stats” package (v4.3.1) [29] in the RStudio platform. Significance levels were denoted as follows: *, *P* < 0.05; **, *P* < 0.01; ***, *P* < 0.001. A level of *P* < 0.05 was deemed statistically significant. To evaluate the equality of variances, Levene's test was used. Data in the figures are presented as mean ± SEM.

## Results

### Aromatase is expressed in the trigeminal ganglion in a sex-dependent manner and aging modifies its expression, inverting sex differences

**Fig. 1:**
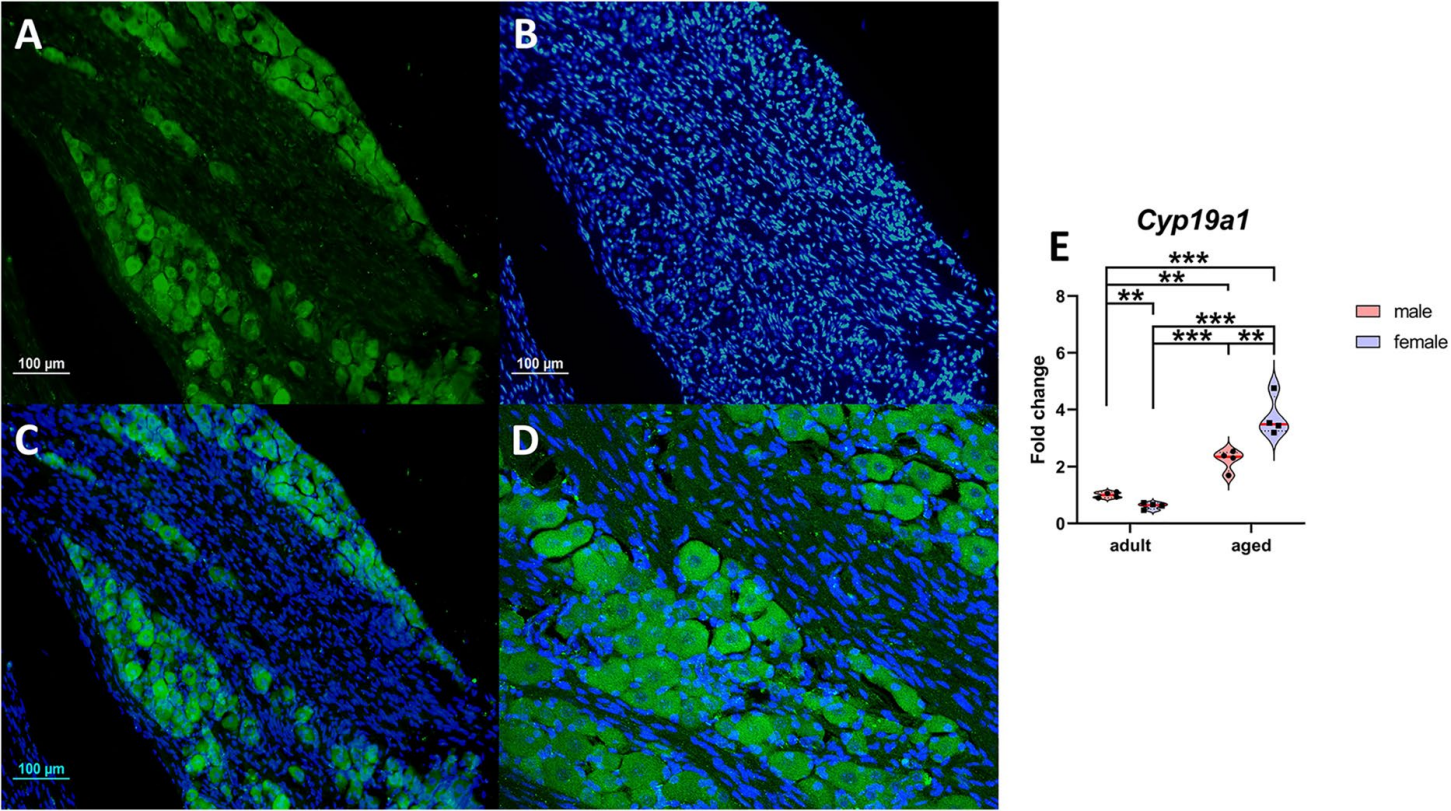
Aromatase expression in the mouse trigeminal ganglion. Representative examples of aromatase immunofluorescence in the mouse trigeminal ganglion and quantification of gene expression in young adult and aged male and female mice. **A**, aromatase marker in the three portions of the trigeminal ganglion. **B**, Trigeminal ganglion cell nuclei labeled with DAPI. **C**, Merge. **D**, Magnification showing aromatase labeling exclusively in the soma of primary sensory neurons, with satellite glial cells not immunoreactive. **E**, Expression of aromatase. Male animals (*n*=4) and female animals (*n*=4). Data are represented as individual values ± SEM. The red line shows mean values. **^,^ *** Significant differences (**p<0.01; ***p<0.001). Scale bar, 100 μm

Aromatase immunoreactivity was identified in primary sensory neurons within the ophthalmic, maxillary, and mandibular subdivisions of the trigeminal ganglion. Immunofluorescence employing the aromatase antibody was exclusively observed in the soma of primary sensory neurons, with satellite glial cells displaying no immunoreactivity (Fig. 1). The absence of immunoreactivity was noted when the primary antibody was omitted (data not shown). Immunoreactivity was exhibited in small, medium, and large primary sensory neurons with a similar staining intensity. This immunoreactivity was confined to the cytoplasm, while cell nuclei remained negative.

Quantification of the *Cyp19a1* gene expression was performed in the trigeminal ganglion of young adult and aged male and female mice. Two-way ANOVA revealed a significant effect of age [*F*_(1,12)_=117.4; *P*<0.0001], of gonadal sex [*F*_(1,12)_=7.829; *P*=0.0161], and the interaction between age and gonadal sex [*F*_(1,12)_=22.02; *P*=0.0005]. Fisher’s multiple comparison tests are presented in Fig. 1.E. Specifically, older animals (27 months of age) exhibited increased *Cyp19a1* expression compared to young adult animals (*P*<0.0001), with older females demonstrating higher expression than older males (*P*<0.01). *Cyp19a1* expression was higher in older females compared to young adult females (*P*<0.0001) and older males (*P*=0.0011), and higher in older males compared to young adult males (*P*<0.0001) (Fig. 1.E).

**Fig. 2:**
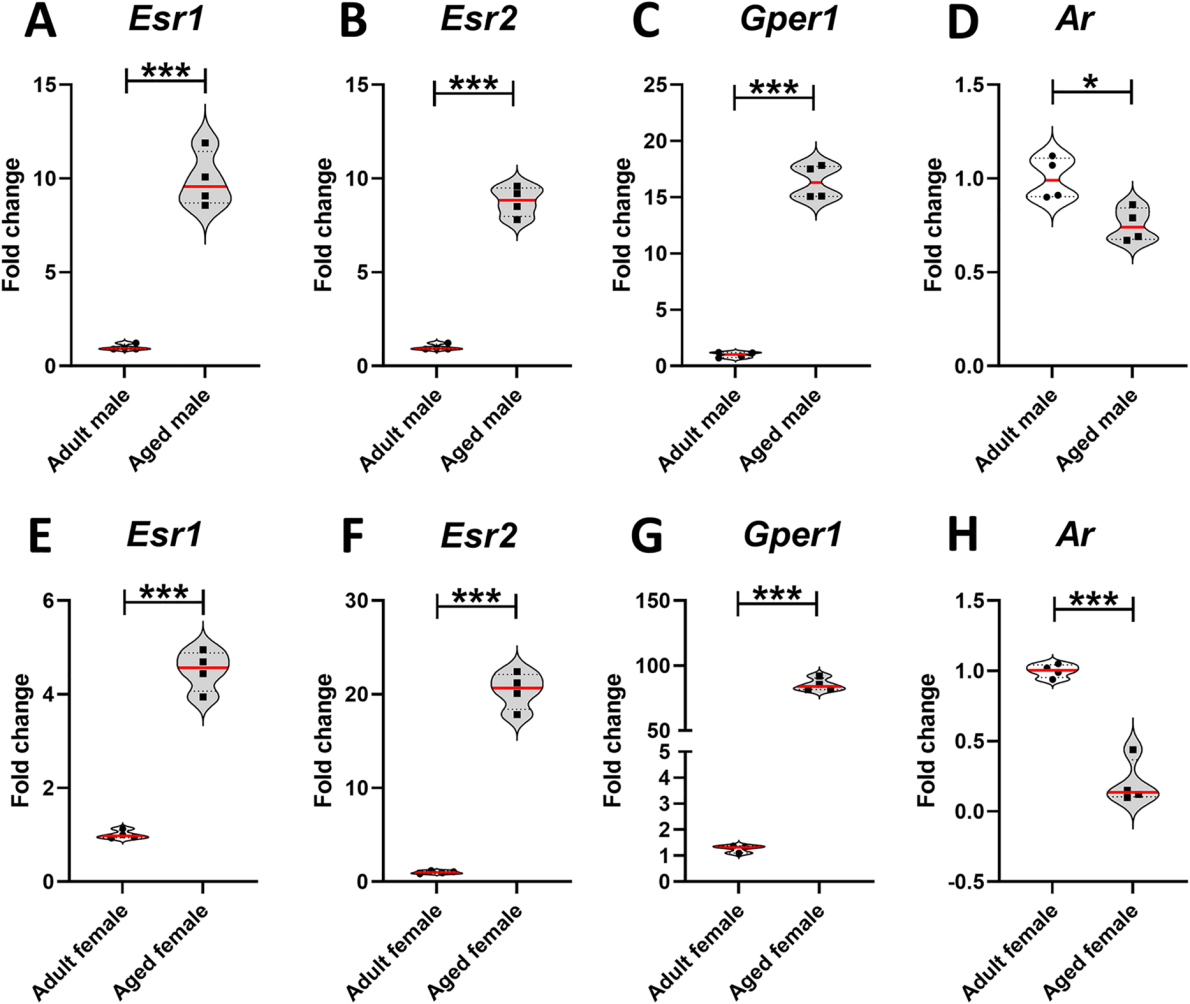
Aging modifies the expression of Cyp19a1 in the trigeminal ganglion. Effect of aging on the aromatase expression in the trigeminal ganglion of aged male and female mice. Comparisons of Cyp19a1 expression levels in young adult and aged male and female animals. Young adult males (*n*=4), young adult females (*n*=4), older males (*n*=4), and older females (*n*=4). Data are represented as individual values ± SEM. The red line shows mean values. *, *** Significant differences (**p<0.01; ***p<0.001)

As a result of the changes in *Cyp19a1* expression with aging, sex differences were inverted in older animals compared to younger ones. Consequently, *Cyp19a1* expression was higher in young males than in young females (Fig. 2A); however, in older animals, *Cyp19a1* expression was elevated in females (Fig. 1.E).

### Aging modifies the expression of *Esr1, Esr2, Gper1 and Ar* in the trigeminal ganglion

Older mice exhibited a significantly higher expression of genes encoding for ERs in the trigeminal ganglion compared to young animals (Fig. 2A-C and E-G). Male animals displayed increased expression of *Esr1* [*t*=12.07, df=6; *P*<0.0001], *Esr2* [*t*=19.31, df=6; *P*<0.0001], and *Gper1* [*t*=20.30, df=6; *P*<0.0001]. Similar results were obtained for female animals, with higher expression levels of *Esr1* [*t*=15.93, df=6; *P*<0.0001], *Esr2* [*t*=19.82, df=6; *P*<0.0001], and *Gper1* [*t*=29.14, df=6; *P*<0.0001] higher in aged animals compared to adult animals.

In contrast to the effects on genes encoding for ERs, aging resulted in a significant decrease in the expression of *Ar* (Fig. 2D and H). Both male [*t*=3.469, df=6; *P*<0.0133] and female [*t*=9.585, df=6; *P*<0.0001] animals exhibited reduced expression of *Ar*.

### Aging reverses sex differences in the expression of *Esr1, Esr2, Gper1, and Ar* in the trigeminal ganglion

**Fig. 3:**
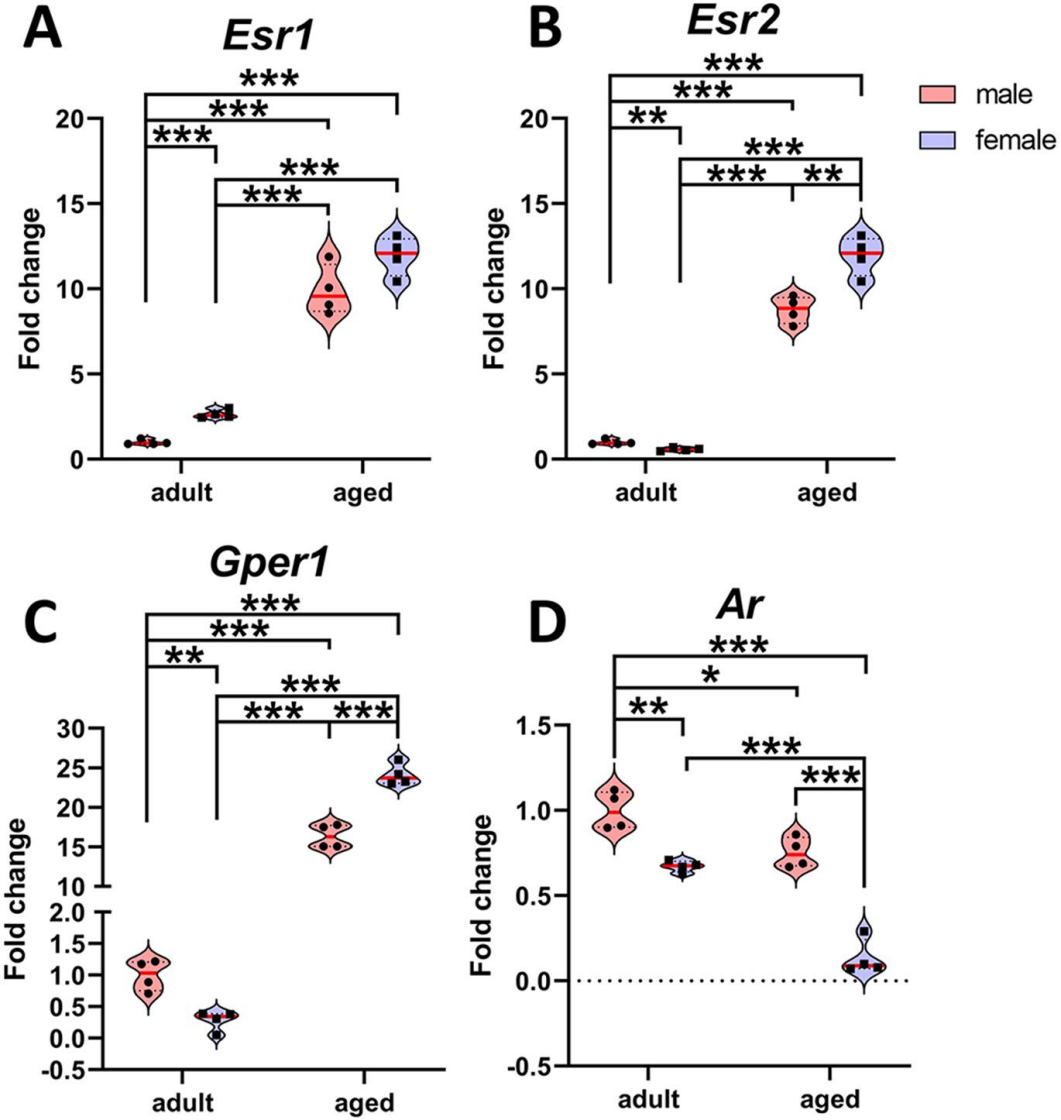
Aging modifies the sex differences in the expression of *Esr1, Esr2, Gper1*, and *Ar* in the trigeminal ganglion. Sex differences in estrogen and androgen receptors’ expression in the trigeminal ganglion of young adult and aged male and female mice. **A**, *Esr1* (ERα) mRNA levels. **B**, *Esr2* (ERβ) mRNA levels. **C**, *Gper1* (GPER) mRNA levels. **D**, *Ar* (AR) mRNA levels in young adult aged male and female animals. Aged male animals (*n*=4) and aged female animals (*n*=4). Data are represented as values distribution ± SEM, the red line shows mean values. *^,^ **^,^ *** Significant differences (*p<0.05; **p<0.01; ***p<0.001)

Due to the aforementioned changes with aging, the sex differences in the expression levels of *Esr1, Esr2, Gper1, and Ar* were assessed in the trigeminal ganglion of young adults and aged male and female mice. Two-way ANOVA revealed a significant effect of age on the expression of *Esr1* [*F*_(1,12)_=373.410; *P*<0.0001], *Esr2* [*F*_(1,12)_=745.06; *P*<0.0001], *Gper1* [*F*_(1,12)_=1462.41; *P*<0.0001] and *Ar* [*F*_(1,12)_=75.13; *P*<0.0001]. Furthermore, gonadal sex displayed a significant effect on *Esr1* [*F*_(1,12)_=15.263; *P*=0.002], *Esr2* [*F*_(1,12)_=15.33; *P*=0.002], *Gper1* [*F*_(1,12)_=47.05; *P*<0.0001] and *Ar* [*F*_(1,12)_=107.07; *P*<0.0001]. Interaction effects between aging and gonadal sex were detected for *Esr2* [*F*_(1,12)_=26.00; *p*=0.0002], *Gper1* [*F*_(1,12)_=68.19; *P*<0.0001] and *Ar* [*F*_(1,12)_=10.12; *P*= 0.007], whereas no interaction was found for *Esr1* [*F*_(1,12)_=0.163; *P*= 0.693]. Fisher’s multiple comparison tests are shown in Fig. 3.

In aged animals, the expression levels of Esr1, Esr2, and Gper1 were markedly increased, with females demonstrating the highest values. This pattern reverses the trend observed in younger subjects, where males exhibited elevated levels. Conversely, Ar expression levels experienced a substantial decline in aging mice, a trend more consistently apparent in female subjects.

### FCG model reveals that sex differences depend on gonadal sex and are not determined by sex chromosome complement

The FCG model enables the determination of whether the origin of sex differences in a specific trait depends on either gonadal sex, sex chromosome complement, or a combination of both factors. This model produces four types of animals: XX and XY mice with testes, and XX and XY mice with ovaries. Comparisons between animals possessing the same type of gonad allow the assessment of sex chromosome influence, while evaluations involving animals with the same sex chromosome complement facilitate the assessment of the influence of gonadal endowment.

**Fig. 4:**
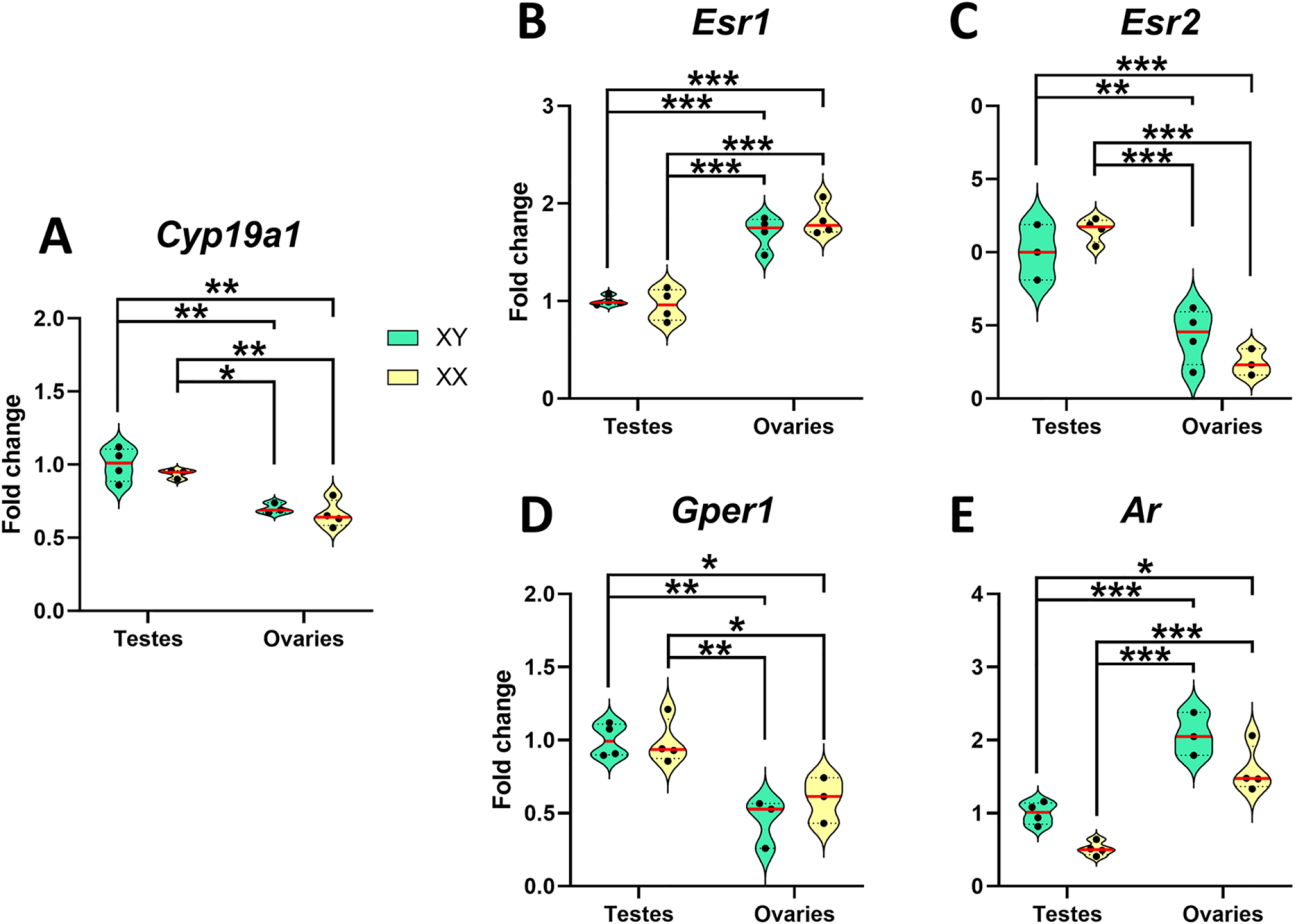
Sex differences in the expression of *Cyp19a1, Esr1, Esr2, Gper1,* and *Ar* depend on gonadal sex and not on sex chromosome complement. Analysis of the influence of gonadal sex and chromosomal complement in the expression of estrogen and androgen receptors in the trigeminal ganglion using the FCG model. **A**, *Cyp19a1* (Aromatase). **B**, *Esr1* (ERα) mRNA levels. **C**, *Esr2* (ERβ) mRNA levels. **D**, *Gper1* (GPER) mRNA levels. **E**, *Ar* (AR) mRNA levels in FCG animals. XY males (*n*=4), XY females (*n*=4), XX males (*n*=4), and XX females (*n*=4). Data are represented as individual values ± SEM. The red line shows mean values. *^,^ **^,^ *** Significant differences (*p<0.05; **p<0.01; ***p<0.001)

Analysis of androgen and estrogen receptor expression and aromatase in FGC animals revealed a significant effect of gonadal endowment (Fig. 4). Animals with testes demonstrated higher expression of *Cyp19a1*, *Esr2,* and *Gper1* than those with ovaries (Fig. 4A, C and D), while animals with ovaries exhibited higher expression levels of *Esr1* and *Ar* than those with testes (Fig. 4B and E), regardless of sex chromosome complement.

Two-way ANOVA revealed a significant effect of gonads on the expression of *Cyp19a1* [*F*_(1,12)_=40.80; *P*<0.0001], *Esr1* [*F*_(1,12)_=116.2; *P*<0.0001], *Esr2* [*F*_(1,12)_=86.99; *P*<0.0001], *Gper1* [*F*_(1,12)_=34.89; *P*=0.0001] and *Ar* [*F*_(1,12)_=79.92; *P*<0.0001]. Additionally, sex chromosome complement displayed a significant effect on *Ar* expression [*F*_(1,12)_=16.53; *P*=0.0019], but not on *Cyp19a1* [*F*_(1,12)_=1.310; *P*=0.2791], *Esr1* [*F*_(1,12)_=0.3385; *P*=0.5714], *Esr2* [*F*_(1,12)_=0.03360; *P*=0.8582], or *Gper1* [*F*_(1,12)_=0.6596; *P*=0.4356] expression.

No interaction effects between gonadal sex and sex chromosome complement were detected for *Cyp19a1* [*F*_(1,12)_=0.06679; *P*=0.8013], *Esr1* [*F*_(1,12)_=1.276; *P*=0.2808], *Esr2* [*F*_(1,12)_=4.543; *P*=0.0589], *Gper1* [*F*_(1,12)_=1.023; *P*=0.3356] and *Ar* [*F*_(1,12)_=1.205; *P*=0.9973]. Fisher’s multiple comparison tests are shown in Fig. 4.

### Significant positive correlation found between *Cyp19a1* expression and *Esr1, Esr2, and Gper1* expression in trigeminal ganglion

**Fig. 5:**
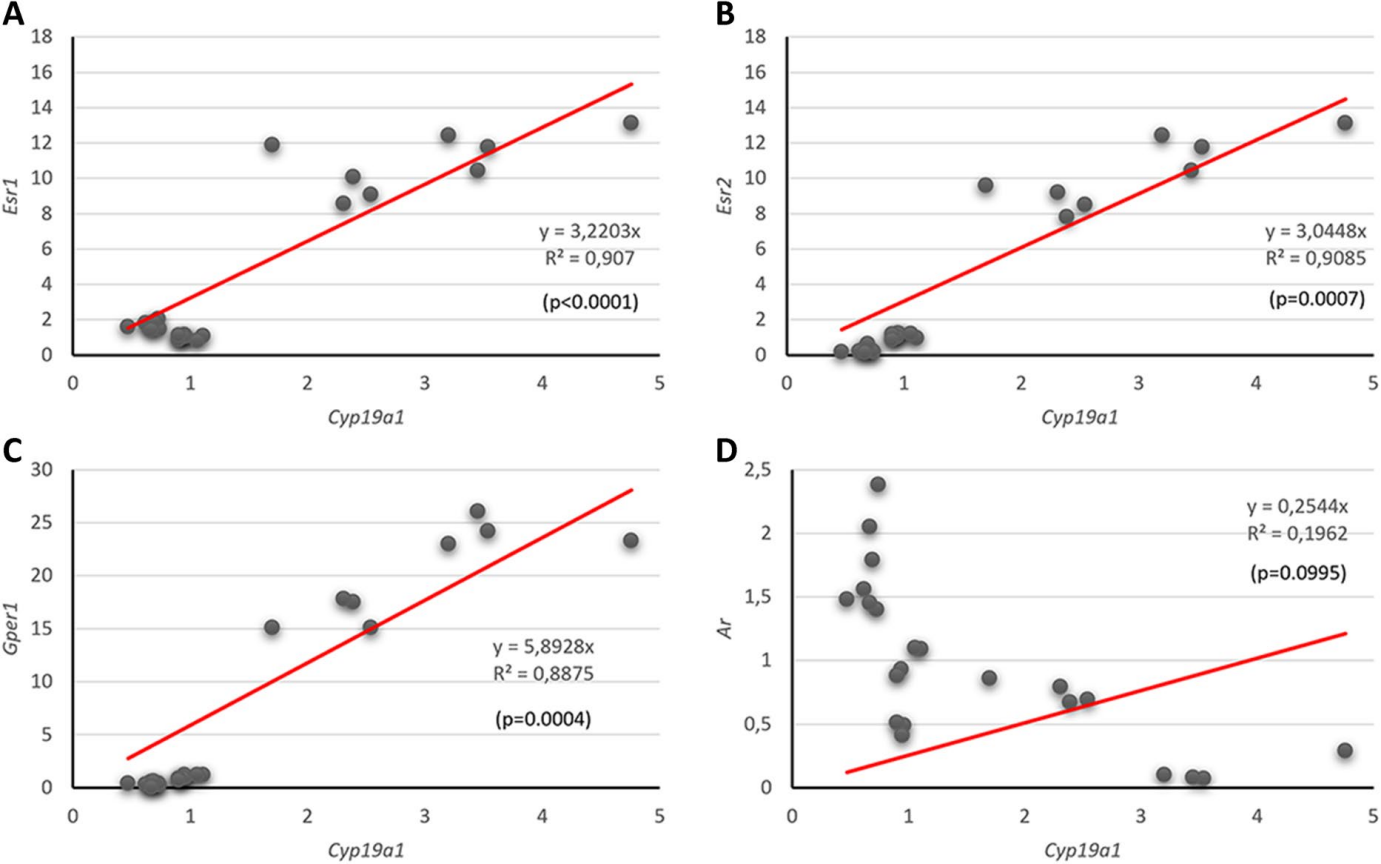
*Cyp19a1* expression significantly and positively correlated with the expression of *Esr1, Esr2,* and *Gper1* in the trigeminal ganglion. Correlation of the levels of aromatase and estrogen and androgen receptors’ expression. **A**, Correlation with *Ers1* (ERα); **B**, Correlation with *Ers2* (ERβ); **C**, Correlation with *Gper1* (GPER); **D**, Correlation with *Ar* (AR)

To investigate whether individual variations in the expression of the gene encoding the enzyme involved in estradiol synthesis (*Cyp19a1* were associated with variations in the expression of genes encoding proteins participating in estradiol signaling, we examined the correlation between individual values of *Cyp19a1* expression and those of *Esr1*, *Esr2*, and *Gper1* in animals from all experimental groups (see Fig. 5).

As illustrated in Fig. 5, significant positive correlations were observed between *Cyp19a1* expression values and *Esr1* [Fig. 5A; *r*=0.95; *R*^2^=0.970; *p*=0.0001], *Esr2* [Fig. 5B; *r*=0.98; *R*^2^= 0.9085; *p*=0.0007] and *Gper1* [Fig. 5C; *r*=0.98; *R*^2^=0.8875; *p*<0.0004] expression values*.* Conversely, a non-significant correlation was demonstrated between *Cyp19a1* expression values and *Ar* [Fig. 5D; *r*=-0.73; *R*^2^=0.1962; *p*=0.0995] expression values.

### Significant positive correlation found between Age and *Esr1, Esr2, Gper1, and Cyp19a1* expression in the trigeminal ganglion of both male and female animals

**Fig. 6:**
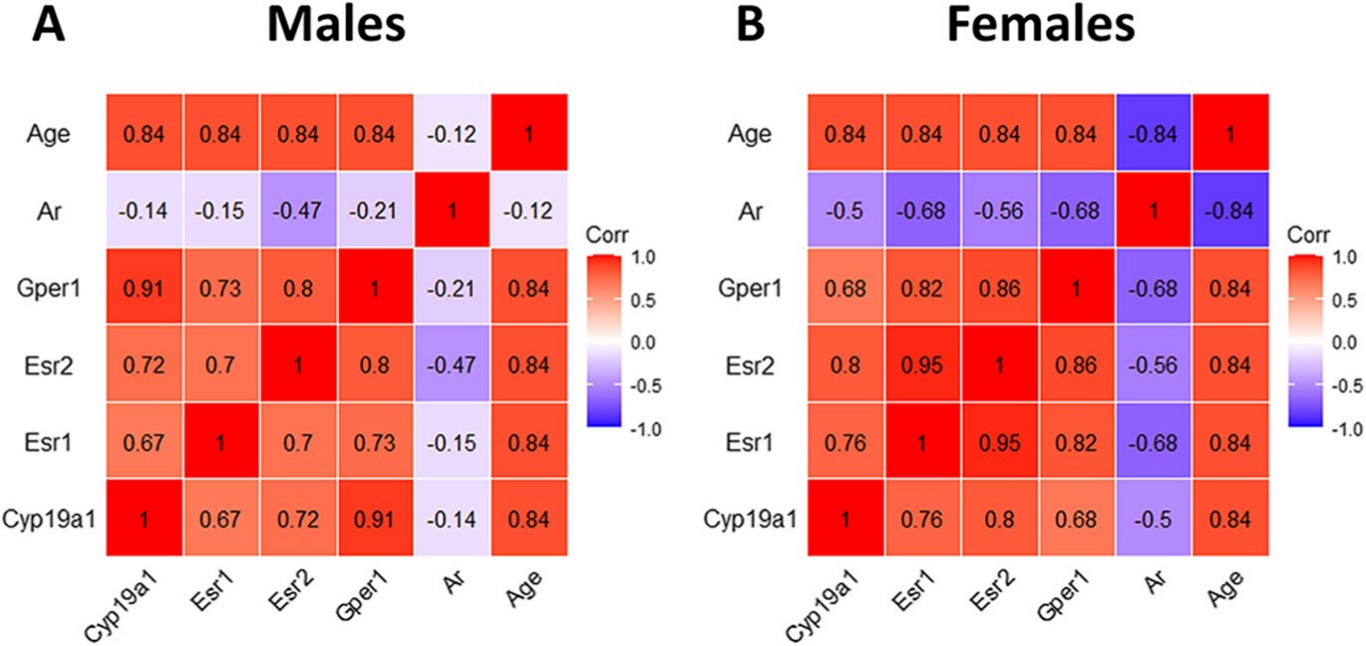
Age significantly and positively correlated with the expression of *Esr1, Esr2,* and *Gper1* in the trigeminal ganglion. Correlation of individual values of *Cyp19a1, Esr1, Esr2, Gper1,* and *Ar* expression at different ages. **A**, Correlation in male animals; **B**, Correlation in female animals

To investigate whether individual variations in the expression of the gene encoding the enzyme involved in estradiol synthesis (*Cyp19a1)* and estradiol and androgen receptor signaling were associated with variations in age, we examined with Spearman's rank correlation coefficient (ρ) the correlation between individual values of *Cyp19a1, Esr1*, *Esr2*, *Gper1 and Ar* expression at different age in male (Fig. 6A) and female (Fig. 6B) animals, respectively.

As illustrated in Fig. 6A, male animals showed significant positive correlations between age and *Cyp19a1* [*ρ*=0.84, *P*=0.001]*, Esr1* [*ρ*=0.84, *P*=0.001]*, Esr2* [*ρ*=0.84, *P*=0.001]*,* and *Gper1* [*ρ*=0.84, *P*=0.001] expression and no correlation with *Ar* values [*ρ*=-0,12, *P*=0.72]. Similarly, female animals showed significant positive correlations between age and *Cyp19a1* [*ρ*=0.84, *P*=0.001]*, Esr1* [*ρ*=0.84, *P*=0.001]*, Esr2* [*ρ*=0.84, *P*=0.001]*,* and *Gper1* [*ρ*=0.84, *P*=0.001] but significant negative correlation with *Ar* values [*ρ*=-0.84, *P*=0.001] (Fig. 6B).

## Discussion

Prior research has demonstrated that inhibiting aromatase increases excitability in small and medium-diameter dorsal root ganglion primary sensory neurons of male rats, suggesting a role for aromatase activity in pain perception regulation [21]. Aromatase immunoreactivity has indeed been identified in primary sensory neurons, although not in satellite cells of the dorsal root ganglia [30]. A similar pattern of aromatase immunoreactivity was observed in the trigeminal ganglion in the current study. However, aromatase immunoreactivity was detected in sensory neurons irrespective of diameter, implying that neuroestradiol could be involved in modulating all sensory modalities of the trigeminal nerve, rather than exclusively pain transmission. In line with previous reports on the expression of ERs in the sensory ganglia of rodents [8, 10, 14], our study detected the expression of *Esr1*, *Esr2,* and *Gper1*, genes encoding ERs, in the trigeminal ganglion. This suggests that neuroestradiol, produced by aromatase-positive trigeminal sensory neurons, may directly act on ERs expressed in the ganglion.

We also investigated the expression of *Ar* in the trigeminal ganglion, as aromatase indirectly affects AR signaling by reducing testosterone levels. The detection of *Ar* in the trigeminal ganglion supports previous findings in adult rats [31–33].

The elevated expression of *Cyp19a1* observed in males in our study implies that testosterone conversion to estradiol is enhanced in this sex. Since estradiol is known to decrease pain perception through actions on primary sensory neurons [8–10], sex differences in aromatase expression may contribute to the observed sex differences in trigeminal neuralgia [34, 35] by increasing neuroestradiol production in male ganglia.

Moreover, observed sex differences in the expression of *Esr1* (higher in females), *Esr2,* and *Gper1* (both higher in males) could potentially create disparities in estrogen signaling in primary sensory neurons, thereby contributing to sex differences in pain perception. In contrast, testosterone, by acting on ARs [35], leads to a decrease in pain perception. Furthermore, AR binding sites have been identified in the promoter of cannabinoid type 1 (CB1) receptors [31], which play a role in modulating analgesia within the trigeminal ganglion [31, 36]. Thus, the higher *Ar* expression detected in the female trigeminal ganglion could also contribute to sex differences in trigeminal pain transmission.

Sex differences in aromatase expression have been observed in various regions of the nervous system and are influenced by perinatal sex hormone levels [37]. Moreover, the sex chromosome complement has been implicated in generating sex differences in some brain structures, such as the anterior amygdala and the stria terminalis [28] in some brain structures, such as the anterior amygdala and the stria terminalis. In order to investigate the mechanisms driving sex differences in *Cyp19a1* in the trigeminal ganglion, we utilized the four-core genotype (FCG) model, allowing for the assessment of the effects of ovarian hormones, sex chromosome complement, or their interaction in determining mouse traits [23].

Our findings indicate that sex differences in C*yp19a1* expression within the mouse trigeminal ganglion are solely dependent on sex hormones, with the presence of testes resulting in higher expression levels. This suggests that elevated androgen levels in males, acting on AR or ERβ [28] lead to higher aromatase expression in the male trigeminal ganglion.

Our research also uncovers sex differences in the expression of genes involved in estrogenic and androgenic signaling within the trigeminal ganglion. Employing the FCG model, we observed that the presence of the XY chromosome complement enhances *Ar* expression in both phenotypically male and female animals, in comparison to those with the XX complement. In contrast, the sex chromosome complement did not impact *Esr1*, *Esr2*, and *Gper1* expression levels, the genes encoding ERs. Furthermore, we discovered that, despite the effect of sex chromosome complement on Ar expression, sex differences in the expression of genes involved in androgen and estrogen signaling were exclusively determined by the gonadal sex.

Sex differences in the nervous system that depend on gonadal sex may be affected by fluctuations in sex hormone levels during an individual's lifespan, including aging. This may be the case for sex differences in pain perception [38], which tend to demonstrate higher pain sensitivity in women. Pain threshold increases with age [39] and aging-related changes that may contribute to a decline in pain perception have been detected in somatosensory neurons of the trigeminal ganglion [40]. In line with previous findings on changes in ER and AR expression in various regions of the nervous system of older animals [41–43], we observed increased expression of genes encoding ERs and decreased expression of the gene encoding AR in aged animals of both sexes. This suggests that aging alters the actions of ovarian and testicular hormones on trigeminal ganglion function.

Aging not only affects the expression of AR and ERs in the nervous system but also impacts aromatase expression. Indeed, changes in aromatase expression have been reported in specific brain regions of older individuals, such as the cerebral cortex and the hippocampus, where an increase in aromatase availability or immunoreactivity has been identified [42, 44]. Our results suggest that this may also be the case for the trigeminal ganglion, where we found that *Cyp19a1* expression increases with age in both sexes.

Regression and correlation analyses of individual data from all animals included in the current study indicate a possible coregulation of the expression of the enzyme involved in estradiol synthesis and the expression of ERs in the trigeminal ganglion, as demonstrated in other systems [45]. This may explain the parallel increase in the expression of Cyp19a1, Esr1, Esr2, and Gper1 in the trigeminal ganglion of older animals.

Given that neuroestradiol, acting on ERs [22], decreases nociception, the increased expression of *Cyp19a1*, *Esr1*, *Esr2,* and *Gper1* in aged animals may potentiate estrogenic signaling, reducing pain transmission by sensory neurons. However, it must be noted that the decrease in *Ar* in older animals could have the opposite effect, increasing pain perception [34, 35] Fanton et al., 2017. Additionally, another consequence of the changes in the expression of *Cyp19a1* and the genes coding for ERs and AR in the trigeminal ganglion with age is that the direction of sex differences in expression patterns observed in young animals becomes inverted in older animals. The functional implications of this inversion for sex differences in pain perception have yet to be determined.

Future research should involve translational studies to confirm if the observed patterns in mice also occur in humans. Investigating the expression of aromatase, estrogen receptors (ERs), and androgen receptors (ARs) in human trigeminal ganglia and correlating these findings with clinical pain conditions can provide valuable insights into the relevance of these molecular changes in human pain perception and may represent possible therapeutic interventions targeting aromatase and estrogen receptors for trigeminal pain management.

Study Limitations: Primarily, this research is limited by the use of animal models, specifically mice, to study complex biological processes. Although the genetic homology between humans and mice provides a valuable framework for understanding mechanisms under controlled experimental settings, the intrinsic anatomical and physiological differences between the species could potentially limit the extrapolation of the findings to humans. In clinical contexts, trigeminal neuralgia is more frequently observed in older adults and presents a gender bias, being more prevalent in women, which is mirrored in our study's findings in mice. While this underscores the potential role of neuroestradiol pathways in the modulation of pain, further studies are needed to confirm their relevance and translate these insights into the human context.

## Conclusions

In conclusion, we here demonstrate that aromatase, the enzyme involved in the synthesis of neuroestradiol, is expressed in primary sensory neurons at the very entrance of the trigeminal sensory pathway and that this expression is altered in older individuals. The existence of sex chromosome-independent sex differences in the expression of aromatase and of the genes encoding ERs indicate that neuroestradiol synthesis and signaling in the trigeminal ganglion is affected by gonads. Furthermore, the observed changes with aging in aromatase and ER expression suggest that the modulation exerted by neuroestradiol on the trigeminal somatosensory input, including pain, is altered in older male and female individuals in a sex-specific manner.

## Data Availability

Data are available on request from the authors. The data that support the findings of this study are available from the corresponding author upon reasonable request.

## References

[1] McIntyre S, Nagi SS, McGlone F, Olausson H. The Effects of Ageing on Tactile Function in Humans. Neuroscience. 2021;464:53–8. 10.1016/j.neuroscience.2021.02.015. 33607227 10.1016/j.neuroscience.2021.02.015

[2] Giovannini S, Coraci D, Brau F, Galluzzo V, Loreti C, Caliandro P, Padua L, Maccauro G, Biscotti L, Bernabei R. Neuropathic Pain in the Elderly. Diagnostics (Basel). 2021;11(4):613. 10.3390/diagnostics11040613. 33808121 10.3390/diagnostics11040613 PMC8066049

[3] Svedung Wettervik T, Snel D, Kristiansson P, Ericson H, Abu HS. Incidence of trigeminal neuralgia: A population-based study in Central Sweden. Eur J Pain. 2023;27(5):580–7. 10.1002/ejp.2081. 36680398 10.1002/ejp.2081

[4] Stompór M, Grodzicki T, Stompór T, Wordliczek J, Dubiel M, Kurowska I. Prevalence of Chronic Pain, Particularly with Neuropathic Component, and Its Effect on Overall Functioning of Elderly Patients. Med Sci Monit. 2019;25:2695–701. 10.12659/MSM.911260. 31018630 10.12659/MSM.911260 PMC6475124

[5] Singh SP, Guindon J, Mody PH, Ashworth G, Kopel J, Chilakapati S, Adogwa O, Neugebauer V, Burton MD. Pain and aging: A unique challenge in neuroinflammation and behavior. Mol Pain. 2023;19:17448069231203090. 10.1177/17448069231203090. 37684099 10.1177/17448069231203090 PMC10552461

[6] Presto P, Mazzitelli M, Junell R, Griffin Z, Neugebauer V. Sex differences in pain along the neuraxis. Neuropharmacology. 2022;210:109030. 10.1016/j.neuropharm.2022.109030. 35331712 10.1016/j.neuropharm.2022.109030 PMC9354808

[7] De Toledo IP, Conti Réus J, Fernandes M, Porporatti AL, Peres MA, Takaschima A, Linhares MN, Guerra E, De Luca Canto G. Prevalence of trigeminal neuralgia: A systematic review. J Am Dent Assoc. 2016;147(7):570–576.e2. 10.1016/j.adaj.2016.02.014. 27017183 10.1016/j.adaj.2016.02.014

[8] Bi RY, Meng Z, Zhang P, Wang XD, Ding Y, Gan YH. Estradiol upregulates voltage-gated sodium channel 1.7 in trigeminal ganglion contributing to hyperalgesia of inflamed TMJ. PLoS One. 2017;12(6):e0178589. 10.1371/journal.pone.0178589. 28582470 10.1371/journal.pone.0178589 PMC5459440

[9] Chen Q, Zhang W, Sadana N, Chen X. Estrogen receptors in pain modulation: cellular signaling. Biol Sex Differ. 2021;12(1):22. 10.1186/s13293-021-00364-5. 33568220 10.1186/s13293-021-00364-5 PMC7877067

[10] Kaur S, Hickman TM, Lopez-Ramirez A, McDonald H, Lockhart LM, Darwish O, Averitt DL. Estrogen modulation of the pronociceptive effects of serotonin on female rat trigeminal sensory neurons is timing dependent and dosage dependent and requires estrogen receptor alpha. Pain. 2022;163(8):e899–916. 10.1097/j.pain.0000000000002604. 35121697 10.1097/j.pain.0000000000002604 PMC9288423

[11] Bereiter DA, Cioffi JL, Bereiter DF. Oestrogen receptor-immunoreactive neurons in the trigeminal sensory system of male and cycling female rats. Arch Oral Biol. 2005;50(11):971–9. 10.1016/j.archoralbio.2005.03.010. 15893734 10.1016/j.archoralbio.2005.03.010

[12] Puri V, Cui L, Liverman CS, Roby KF, Klein RM, Welch KM, Berman NE. Ovarian steroids regulate neuropeptides in the trigeminal ganglion. Neuropeptides. 2005;39(4):409–17. 10.1016/j.npep.2005.04.002. 15936815 10.1016/j.npep.2005.04.002

[13] Liverman CS, Brown JW, Sandhir R, McCarson KE, Berman NE. Role of the oestrogen receptors GPR30 and ERalpha in peripheral sensitization: relevance to trigeminal pain disorders in women. Cephalalgia. 2009;29(7):729–41. 10.1111/j.1468-2982.2008.01789.x. 19220308 10.1111/j.1468-2982.2008.01789.x PMC4054707

[14] Warfvinge K, Krause DN, Maddahi A, Edvinsson JCA, Edvinsson L, Haanes KA. Estrogen receptors α, β and GPER in the CNS and trigeminal system - molecular and functional aspects. J Headache Pain. 2020;21(1):131. 10.1186/s10194-020-01197-0. 33167864 10.1186/s10194-020-01197-0 PMC7653779

[15] Evrard HC, Balthazart J. Rapid regulation of pain by estrogens synthesized in spinal dorsal horn neurons. J Neurosci. 2004;24(33):7225–9. 10.1523/JNEUROSCI.1638-04.2004. 15317848 10.1523/JNEUROSCI.1638-04.2004 PMC6729773

[16] Tran M, Kuhn JA, Bráz JM, Basbaum AI. Neuronal aromatase expression in pain processing regions of the medullary and spinal cord dorsal horn. J Comp Neurol. 2017;525(16):3414–28. 10.1002/cne.24269. 28649695 10.1002/cne.24269 PMC6174086

[17] Storman EM, Liu NJ, Wessendorf MW, Gintzler AR. Physical Linkage of Estrogen Receptor α and Aromatase in Rat: Oligocrine and Endocrine Actions of CNS-Produced Estrogens. Endocrinology. 2018;159(7):2683–97. 10.1210/en.2018-00319. 29771302 10.1210/en.2018-00319 PMC6692873

[18] Laroche F, Coste J, Medkour T, Cottu PH, Pierga JY, Lotz JP, Beerblock K, Tournigand C, Declèves X, de Cremoux P, Bouhassira D, Perrot S. Classification of and risk factors for estrogen deprivation pain syndromes related to aromatase inhibitor treatments in women with breast cancer: a prospective multicenter cohort study. J Pain. 2014;15(3):293–303. 10.1016/j.jpain.2013.11.004. 24365325 10.1016/j.jpain.2013.11.004

[19] Fusi C, Materazzi S, Benemei S, Coppi E, Trevisan G, Marone IM, Minocci D, De Logu F, Tuccinardi T, Di Tommaso MR, Susini T, Moneti G, Pieraccini G, Geppetti P, Nassini R. Steroidal and non-steroidal third-generation aromatase inhibitors induce pain-like symptoms via TRPA1. Nat Commun. 2014;5:5736. 10.1038/ncomms6736. 25484020 10.1038/ncomms6736 PMC4268712

[20] Ghorbanpoor S, Garcia-Segura LM, Haeri-Rohani A, Khodagholi F, Jorjani M. Aromatase inhibition exacerbates pain and reactive gliosis in the dorsal horn of the spinal cord of female rats caused by spinothalamic tract injury. Endocrinology. 2014;155(11):4341–55. 10.1210/en.2014-1158. 25105782 10.1210/en.2014-1158

[21] Robarge JD, Duarte DB, Shariati B, Wang R, Flockhart DA, Vasko MR. Aromatase inhibitors augment nociceptive behaviors in rats and enhance the excitability of sensory neurons. Exp Neurol. 2016;281:53–65. 10.1016/j.expneurol.2016.04.006. 27072527 10.1016/j.expneurol.2016.04.006 PMC4877294

[22] Gintzler AR, Storman EM, Liu NJ. Estrogens as arbiters of sex-specific and reproductive cycle-dependent opioid analgesic mechanisms. Vitam Horm. 2019;111:227–46. 10.1016/bs.vh.2019.06.002. 31421702 10.1016/bs.vh.2019.06.002 PMC7136895

[23] Arnold AP. Four Core Genotypes and XY* mouse models: Update on impact on SABV research. Neurosci Biobehav Rev. 2020;119:1–8. 10.1016/j.neubiorev.2020.09.021. 32980399 10.1016/j.neubiorev.2020.09.021 PMC7736196

[24] Itoh Y, Mackie R, Kampf K, Domadia S, Brown JD, O'Neill R, Arnold AP. Four core genotypes mouse model: localization of the Sry transgene and bioassay for testicular hormone levels. BMC Res Notes. 2015;8:69. 10.1186/s13104-015-0986-2. 25870930 10.1186/s13104-015-0986-2 PMC4354741

[25] Llorente R, Marraudino M, Carrillo B, Bonaldo B, Simon-Areces J, Abellanas-Pérez P, Rivero-Aguilar M, Fernandez-Garcia JM, Pinos H, Garcia-Segura LM, Collado P, Grassi D. G Protein-Coupled Estrogen Receptor Immunoreactivity Fluctuates During the Estrous Cycle and Show Sex Differences in the Amygdala and Dorsal Hippocampus. Front Endocrinol (Lausanne). 2020;11:537. 10.3389/fendo.2020.00537. 32849310 10.3389/fendo.2020.00537 PMC7426398

[26] Yague JG, Muñoz A, de Monasterio-Schrader P, Defelipe J, Garcia-Segura LM, Azcoitia I. Aromatase expression in the human temporal cortex. Neuroscience. 2006;138(2):389–401. 10.1016/j.neuroscience.2005.11.054. 16426763 10.1016/j.neuroscience.2005.11.054

[27] Ruiz-Palmero I, Hernando M, Garcia-Segura LM, Arevalo MA. G protein-coupled estrogen receptor is required for the neuritogenic mechanism of 17β-estradiol in developing hippocampal neurons. Mol Cell Endocrinol. 2013;372(1-2):105–15. 10.1016/j.mce.2013.03.018. 23545157 10.1016/j.mce.2013.03.018

[28] Cisternas CD, Cabrera Zapata LE, Arevalo MA, Garcia-Segura LM, Cambiasso MJ. Regulation of aromatase expression in the anterior amygdala of the developing mouse brain depends on ERβ and sex chromosome complement. Sci Rep. 2017;7(1):5320. 10.1038/s41598-017-05658-6. 28706210 10.1038/s41598-017-05658-6 PMC5509695

[29] R Core Team. R: a language and environment for statistical computing. Vienna, Austria: R Foundation for Statistical Computing; 2021. https://www.R-project.org/. Accessed 15 Dec 2021.

[30] Schaeffer V, Meyer L, Patte-Mensah C, Eckert A, Mensah-Nyagan AG. Sciatic nerve injury induces apoptosis of dorsal root ganglion satellite glial cells and selectively modifies neurosteroidogenesis in sensory neurons. Glia. 2010;58(2):169–80. 10.1002/glia.20910. 19565659 10.1002/glia.20910

[31] Lee KS, Asgar J, Zhang Y, Chung MK, Ro JY. The role of androgen receptor in transcriptional modulation of cannabinoid receptor type 1 gene in rat trigeminal ganglia. Neuroscience. 2013;254:395–403. 10.1016/j.neuroscience.2013.09.014. 24055403 10.1016/j.neuroscience.2013.09.014 PMC3870904

[32] Lee KS, Zhang Y, Asgar J, Auh QS, Chung MK, Ro JY. Androgen receptor transcriptionally regulates μ-opioid receptor expression in rat trigeminal ganglia. Neuroscience. 2016;331:52–61. 10.1016/j.neuroscience.2016.06.023. 27320211 10.1016/j.neuroscience.2016.06.023 PMC5498154

[33] Young WJ, Chang C. Ontogeny and autoregulation of androgen receptor mRNA expression in the nervous system. Endocrine. 1998;9(1):79–88. 10.1385/ENDO:9:1:79. 9798734 10.1385/ENDO:9:1:79

[34] Fanton LE, Macedo CG, Torres-Chávez KE, Fischer L, Tambeli CH. Activational action of testosterone on androgen receptors protects males preventing temporomandibular joint pain. Pharmacol Biochem Behav. 2017;152:30–5. 10.1016/j.pbb.2016.07.005. 27461546 10.1016/j.pbb.2016.07.005

[35] Shaefer JR, Khawaja SN, Bavia PF. Sex, Gender, and Orofacial Pain. Dent Clin North Am. 2018;62(4):665–82. 10.1016/j.cden.2018.06.001. 30189989 10.1016/j.cden.2018.06.001

[36] Li ZW, Zhang J, Ouyang CH, Li CY, Zhao FB, Liu YW, Ai YX, Hu WP. Potentiation by WIN 55,212-2 of GABA-activated currents in rat trigeminal ganglion neurones. Br J Pharmacol. 2009;158(8):1904–10. 10.1111/j.1476-5381.2009.00482.x. 19917064 10.1111/j.1476-5381.2009.00482.x PMC2807652

[37] Roselli CE, Klosterman SA. Sexual differentiation of aromatase activity in the rat brain: effects of perinatal steroid exposure. Endocrinology. 1998;139(7):3193–201. 10.1210/endo.139.7.6101. 9645693 10.1210/endo.139.7.6101

[38] Athnaiel O, Cantillo S, Paredes S, Knezevic NN. The Role of Sex Hormones in Pain-Related Conditions. Int J Mol Sci. 2023;24(3):1866. 10.3390/ijms24031866. 36768188 10.3390/ijms24031866 PMC9915903

[39] Lautenbacher S, Peters JH, Heesen M, Scheel J, Kunz M. Age changes in pain perception: A systematic-review and meta-analysis of age effects on pain and tolerance thresholds. Neurosci Biobehav Rev. 2017;75:104–13. 10.1016/j.neubiorev.2017.01.039. 28159611 10.1016/j.neubiorev.2017.01.039

[40] Oto T, Urata K, Hayashi Y, Hitomi S, Shibuta I, Iwata K, Iinuma T, Shinoda M. Age-Related Differences in Transient Receptor Potential Vanilloid 1 and 2 Expression Patterns in the Trigeminal Ganglion Neurons Contribute to Changes in the Palatal Mucosal Heat Pain Sensitivity. Tohoku J Exp Med. 2022;256(4):283–90. 10.1620/tjem.2022.J004. 35296569 10.1620/tjem.2022.J004

[41] Wilson ME, Rosewell KL, Kashon ML, Shughrue PJ, Merchenthaler I, Wise PM. Age differentially influences estrogen receptor-alpha (ERalpha) and estrogen receptor-beta (ERbeta) gene expression in specific regions of the rat brain. Mech Ageing Dev. 2002;123(6):593–601. 10.1016/s0047-6374(01)00406-7. 11850023 10.1016/s0047-6374(01)00406-7

[42] Ishunina TA, Fischer DF, Swaab DF. Estrogen receptor alpha and its splice variants in the hippocampus in aging and Alzheimer's disease. Neurobiol Aging. 2007;28(11):1670–81. 10.1016/j.neurobiolaging.2006.07.024. 17010478 10.1016/j.neurobiolaging.2006.07.024

[43] Wu D, Gore AC. Changes in androgen receptor, estrogen receptor alpha, and sexual behavior with aging and testosterone in male rats. Horm Behav. 2010;58(2):306–16. 10.1016/j.yhbeh.2010.03.001. 20223236 10.1016/j.yhbeh.2010.03.001 PMC2879440

[44] Biegon A. In vivo visualization of aromatase in animals and humans. Front Neuroendocrinol. 2016;40:42–51. 10.1016/j.yfrne.2015.10.001. 26456904 10.1016/j.yfrne.2015.10.001 PMC4783227

[45] Yilmaz MB, Zhao H, Brooks DC, Fenkci IV, Imir-Yenicesu G, Attar E, Akbal E, Kaynak BA, Bulun SE. Estrogen receptor alpha (Esr1) regulates aromatase (Cyp19a1) expression in the mouse brain. Neuro Endocrinol Lett. 2015;36(2):178–82. 26071589

